# Chronic Prostatitis/Chronic Pelvic Pain Syndrome is associated with Irritable Bowel Syndrome: A Population-based Study

**DOI:** 10.1038/srep26939

**Published:** 2016-05-26

**Authors:** Chun-Hou Liao, Herng-Ching Lin, Chao-Yuan Huang

**Affiliations:** 1 Division of Urology, Department of Surgery, Cardinal Tien Hospital, Taipei, Taiwan; 2 College of Medicine, Fu-Jen Catholic University, New Taipei City, Taiwan; 3 Sleep Research Center, Taipei Medical University Hospital, Taipei, Taiwan; 4 School of Public Health, Taipei Medical University, Taipei, Taiwan; 5 Department of Urology, National Taiwan University Hospital, College of Medicine National Taiwan University, Taipei, Taiwan

## Abstract

This study aimed to examine this association by comparing the risk of prior irritable bowel syndrome (IBS) between patients with chronic prostatitis/chronic pelvic pain syndrome (CP/CPPS) and matched controls in Taiwan. Data were retrieved from the Longitudinal Health Insurance Database 2005. This study included 4870 cases with CP/CPPS and 4870 age-matched controls. Conditional logistic regressions were conducted to examine associations of CP/CPPS with previously diagnosed IBS. We found that a total of 753 (7.7%) of the 9740 sampled patients had IBS prior to the index date; IBS was found in 497 (10.2%) cases and in 256 (5.3%) controls. Conditional logistic regression revealed a higher odds ratio (OR) of prior IBS (OR 2.05, 95% CI = 1.75–2.40) for cases than controls. Furthermore, after adjusting for the patients’ monthly income, geographical location, urbanization level, and hypertension and coronary heart disease, the conditional logistic regression analysis indicated that cases were more likely than controls to have prior IBS (OR = 1.96, 95% CI = 1.67–2.29). Furthermore, we found that CP/CPPS was consistently and significantly associated with prior IBS regardless of age group. We concluded that the diagnosis of CP/CPPS was associated with previously diagnosed IBS. Urologists should be aware of the association between CP/CPPS and IBS when treating patients.

Prostatitis is a poorly defined condition and is shown to have a bacterial aetiology in 5 to 10% of cases^1^,^2^,^3^. In the remaining proportion, the symptoms are attributed to “chronic non-bacterial prostatitis” or “chronic prostatitis/chronic pelvic pain syndrome (CP/CPPS)”. However, the potential etiologies and pathogenesis for CP/CPPS still remained unclear.

Over the years, the concomitant presence of unexplained non-urological clinical conditions, such as fibromyalgia, chronic fatigue syndrome, and irritable bowel syndrome (IBS), and some urological illnesses, including chronic pelvic pain, interstitial cystitis, painful bladder syndrome, CP/CPPS, and vulvodynia, have attracted great interest^4^. Of these clinical conditions, IBS is the most common functional gastrointestinal disorder considered to result from multiple factors, including hypersensitivity of the bowel, altered bowel motility, inflammation, and stress^5^. Therefore, CP/CPPS and IBS may share some peculiar features. They are both functional, somatoform disorders with a high worldwide prevalence, and both conditions are defined on the basis of the clinical presentation rather than clear diagnostic markers or findings^6^. A previous case-control study showed that CP/CPPS patients had an average of 2.4 co-morbid disorders including IBS^7^. Pontari *et al.* reported IBS history in 12.8% of patients with CP/CPPS symptoms vs. 2.5% of controls using symptom-based diagnostic criteria^8^. Clemens *et al.* also reported IBS in 22.4% of patients with CP/CPPS using ICD-9 as diagnosed criteria^9^. However, to date, all such studies have relied upon regional samples, or on data from a few selected hospitals or sub-populations of patients, and as such, do not permit unequivocal conclusions.

Therefore, in the present case-control study, we utilized a population-based dataset to examine the association between these conditions by comparing the incidence of prior IBS between subjects with CP/CPPS and matched controls in Taiwan.

## Methods

### Database

The data for this study were retrieved from the Longitudinal Health Insurance Database 2005 (LHID2005). The LHID2005, which was created by Taiwan National Health Research Institute, includes all the original medical claim data and registration files of 1,000,000 individuals, randomly sampled from the 2005 Registry for Beneficiaries (*n* = 25.68 million) of the Taiwan National Health Insurance (NHI) program.

### Selection of Cases and Controls

In selected cases, we first identified 7246 patients who received their first diagnosis of CP/CPPS (ICD-9CM code 601.1) in ambulatory care visits between January 2001 and December 2013. We defined the date of receiving their first diagnosis of CP/CPPS as the index date. As there are no definitive diagnostic tests for diagnosing CP/CPPS, this study only included those patients who had received two or more CP/CPPS diagnoses prior to the index date, with at least one being made by a certified urologist, in order to increase the diagnostic validity of CP/CPPS cases (*n* = 5011). Furthermore, we excluded 141 CP/CPPS patients, who received a diagnosis of prostate cancer, inguinal hernia, interstitial cystitis, or urethritis within one year before and after the index date, in order to eliminate the possibility of misdiagnosis of these diseases with CP/CPPS. Ultimately, 4870 patients with CP/CPPS were included as cases.

Similarly, matched controls (*n* = 4870) were selected from the LHID2005, matched on age, with a stratification basis of ten-years (<30, 30–39, 40–49, 50–59, 60–69, and >69), and index year. The controls were selected by matching them to CP/CPPS cases based on their utilization of medical services in the same index year of that particular case. None of the selected controls had a history of CP/CPPS since the initiation of the NHI program in 1995. We further defined the first use of ambulatory care visits occurring in the index year as the index date for controls. Correspondingly, we confirmed that none of the selected controls had ever received a diagnosis of prostate cancer, inguinal hernia, interstitial cystitis, or urethritis within one year before and after the index date.

### Exposure assessment

IBS cases were identified by a diagnosis of ICD-9-CM code 564.1 in the medical claim during ambulatory care visits. To increase the diagnosis validity, this study only included those IBS patients who had received two or more IBS diagnoses, with at least one being made by a certified gastroenterologist. In addition, we only included cases with at least one diagnosis of IBS prior to the index date.

### Statistical Analysis

The SAS system (SAS System for Windows, Version 8.2, SAS Institute Inc., Cary, NC) was used for statistical analyses. Chi-square tests were performed to examine differences in the monthly income, geographical location, and urbanization level of the patient’s residence between cases and controls. We also compared the distribution of hypertension, diabetes, and coronary heart disease (CHD) between cases and controls. In addition, conditional logistic regressions were conducted to examine association of CP/CPPS with previously diagnosed IBS. The conventional *P *≤ 0.05 was used to assess statistical significance.

## Results

Table 1 presents the distribution of demographic characteristics and medical co-morbidities between cases and controls. After age matching on a 10-year basis, there were significant differences in the monthly income, geographical location, and urbanization level of the patient’s residence between cases and controls (all P < 0.001). In addition, cases were more likely to have co-morbidities of hypertension (38.1% vs. 35.2%, P = 0.004) and CHD (16.8% vs. 13.9%, P < 0.001) than controls.

Table 2 shows the prevalence of prior IBS between cases and controls. A total of 753 (7.7%) out of the 9740 sampled patients had IBS prior to the index date; IBS was found in 497 (10.2%) cases and in 256 (5.3%) controls (P < 0.001). Conditional logistic regression analysis, conditioned on age (10-year basis) and index year, revealed that the odds ratio (OR) of prior IBS was higher for cases than for controls (OR 2.05, 95% CI = 1.75–2.40, P < 0.001). Furthermore, after adjusting for the patients’ monthly income, geographical location, urbanization level, and hypertension and CHD, the conditional logistic regression analysis indicated that cases were more likely to have prior IBS (OR = 1.96, 95% CI = 1.67–2.29) when compared with controls.

## Discussion

Here, we report that the OR of prior IBS was significantly higher (10.2% vs. 5.3%, P < 0.001) for cases than for controls. Furthermore, after adjusting for the patients’ sociodemographic characteristics and co-morbidities, the conditional logistic regression analysis indicated that cases were more likely than controls to have prior IBS (OR = 1.96).

To our knowledge, the present study is the first and largest case-control study utilizing a population-based dataset to examine the association between IBS and CP/CPPS by comparing the incidence of prior IBS between patients with CP/CPPS and matched controls. Clemens *et al.* have previously reported IBS in 22.4% of patients with CP/CPPS using ICD-9 as diagnosed criteria^9^. However, the authors report that only 174 men were recruited from an academic urology practice at a tertiary care facility, and no control group was used. Our results provide more compelling evidence that CP/CPPS is significantly associated with prior IBS. We found that cases were more likely than controls to have co-morbidities of hypertension (38.1% vs. 35.2%, P = 0.004) and CHD (16.8% vs. 13.9%, P < 0.001). This finding was consistent with that from a previous case-control study of men with CP/CPPS, where cases had a significantly higher prevalence of self-reported cardiovascular disease than age matched controls^10^. In men, a previous heart attack was independently associated with worse CP/CPPS symptoms^9^. Although speculative, increased autonomic nervous system activity may contribute to the two conditions. However, further mechanistic studies are needed before any significant conclusions can be drawn.

Over the years, many investigations on chronic pelvic pain (CPP) have focused on peripheral-end-organ mechanisms, such as inflammatory or infective conditions. However, both, animal and clinical studies have indicated that many of the mechanisms for CPPS are based within the central nervous system (CNS)^11^. Central sensitization is responsible for a decrease in the threshold and an increase in the response duration and magnitude of dorsal horn neurons^12^. As a result, sensitization increases signalling to the CNS and amplifies what is perceived from a peripheral stimulus. In visceral hyperalgaesia, stimuli that are normally sub-threshold may result in a sensation of fullness and a need to void the bladder or to defecate^1^. Normally perceived stimuli may be interpreted as pain, and stimuli that are normally noxious may be magnified (true hyperalgaesia), with an increased perception of pain^1^. Viscero-visceral hyperalgaesia is thought to be due to two or more organs with converging sensory projections and central sensitization. There is an anatomic overlap of peripheral nerves to the bowel and the urinary system^13^. Irritation of the bowel with an inflammatory agent also leads to a decrease in the interval to contraction during bladder filling^14^.

Functional Magnetic Resonance Imaging (FMRI) has indicated that the psychological modulation of visceral pain likely involves multiple pathways^15^. Although our results showed that CP/CPPS is associated with previously diagnosed IBS, significant differences in specific anatomical regions have been reported in CP/CPPS patients but not in IBS patients. The MAPP network neuroimaging study revealed that patients with CPPS have extensive microstructural differences within the brain, which are localized to regions associated with the perception and integration of sensory information and pain modulation^16^. These unique changes appear to be a consequence of longstanding pain. The pathophysiology of the association between IBS and CP/CPPS may be associated with the peripheral nervous system rather than the CNS. Further studies are needed to clarify the underlying mechanisms that link IBS and CP/CPPS.

The strength of the present study lies in the large population-based database used, which allowed us to avoid pitfalls, such as selection biases, which are otherwise unavoidable in studies that use data taken from voluntary registries or hospital-referred study patients. The use of a population-based dataset in our study also avoids the effects of a recall bias.

Nevertheless, some limitations of the present study need to be addressed. First, although relying on data derived from administrative claims data may have allowed us to avoid recall bias, such data may be less accurate than diagnoses made according to the standardized criteria of validated questionnaires. To avoid mistaken diagnoses, only patients who had been diagnosed at least twice with both, IBS and CP/CPPS, with each condition being diagnosed at least once by a certified specialist, were selected.

## Conclusions

We concluded that the diagnosis of CP/CPPS is associated with previously diagnosed IBS. Urologists should be aware of the association between CP/CPPS and IBS in subjects suffering from IBS.

## Additional Information

**How to cite this article**: Liao, C.-H. *et al.* Chronic Prostatitis/Chronic Pelvic Pain Syndrome is associated with Irritable Bowel Syndrome: A Population-based Study. *Sci. Rep.* **6**, 26939; doi: 10.1038/srep26939 (2016).

## Figures and Tables

**Table 1:** Demographic characteristics of patients with chronic prostatitis/chronic pelvic pain syndrome and controls in Taiwan (n = 9740).

Variable	Patients with CP/CPPS (n = 4870)		Controls (n = 4870)		*P* value
	Total No.	%	Total No.	%	
Age (years)					>0.999
<30	338	6.9	338	6.9	
30–39	682	14.0	682	14.0	
40–49	896	18.4	896	18.4	
50–59	962	19.8	962	19.8	
60–69	876	18.0	876	18.0	
>69	1116	22.9	1116	22.9	
Monthly Income					<0.001
NT$0-15,840	1736	35.7	1866	38.3	
NT$15,841–25,000	1331	27.3	1584	32.5	
≥NT$25,001	1803	37.0	1420	29.2	
Geographic region					<0.001
Northern	2825	58.0	2323	47.7	
Central	871	17.9	1157	23.8	
Eastern	1071	22.0	1273	26.1	
Southern	103	2.1	117	2.4	
Urbanization level					<0.001
1 (most urbanized)	1886	38.7	1412	29.0	
2	1272	26.1	1352	27.8	
3	662	13.6	833	17.1	
4	535	11.0	694	14.2	
5 (least urbanized)	515	10.6	579	11.9	
Diabetes	758	15.6	708	14.5	0.157
Hypertension	1854	38.1	1716	35.2	0.004
Coronary heart disease	817	16.8	675	13.9	<0.001

**Table 2:** Prevalence and odds ratios for irritable bowel syndrome among the sampled patients.

Presence of prior irritable bowel syndrome	Total (*n* = 9740)		Patients with chronic prostatitis/chronic pelvic pain syndrome (*n* = 4870)		Controls (*n* = 4870)	
	*n*, %		*n*, %		*n*, %	
Yes	753	7.7	497	10.2	256	5.3
Crude OR (95% CI)	–		2.05*** (1.75–2.40)		1.00	
Adjusted OR^a^ (95% CI)	–		1.96*** (1.67–2.29)		1.00	

Notes: OR = odds ratio; OR was calculated by conditional logistic regression which was conditioned on age group; ^a^Adjustments are made for patient’s monthly income, geographic region, urbanization level, coronary heart disease and hypertension; ***indicates P < 0.001.
